# The Total Deviation Index estimated by Tolerance Intervals to evaluate the concordance of measurement devices

**DOI:** 10.1186/1471-2288-10-31

**Published:** 2010-04-08

**Authors:** Geòrgia Escaramís, Carlos Ascaso, Josep L Carrasco

**Affiliations:** 1 Bioestadística, Departament de Salut Pública, Universitat de Barcelona, Catalonia, Spain; 2 CIBER en Epidemiología y Salud Pública (CIBERESP), Spain; 3 Centre de Salut Internacional, Institut d'Investigacions Biomèdiques August Pi i Sunyer. IDIBAPS, Barcelona, Catalonia, Spain

## Abstract

**Background:**

In an agreement assay, it is of interest to evaluate the degree of agreement between the different methods (devices, instruments or observers) used to measure the same characteristic. We propose in this study a technical simplification for inference about the total deviation index (TDI) estimate to assess agreement between two devices of normally-distributed measurements and describe its utility to evaluate inter- and intra-rater agreement if more than one reading per subject is available for each device.

**Methods:**

We propose to estimate the TDI by constructing a probability interval of the difference in paired measurements between devices, and thereafter, we derive a tolerance interval (TI) procedure as a natural way to make inferences about probability limit estimates. We also describe how the proposed method can be used to compute bounds of the coverage probability.

**Results:**

The approach is illustrated in a real case example where the agreement between two instruments, a handle mercury sphygmomanometer device and an OMRON 711 automatic device, is assessed in a sample of 384 subjects where measures of systolic blood pressure were taken twice by each device. A simulation study procedure is implemented to evaluate and compare the accuracy of the approach to two already established methods, showing that the TI approximation produces accurate empirical confidence levels which are reasonably close to the nominal confidence level.

**Conclusions:**

The method proposed is straightforward since the TDI estimate is derived directly from a probability interval of a normally-distributed variable in its original scale, without further transformations. Thereafter, a natural way of making inferences about this estimate is to derive the appropriate TI. Constructions of TI based on normal populations are implemented in most standard statistical packages, thus making it simpler for any practitioner to implement our proposal to assess agreement.

## Background

In an agreement assay, it is of interest to evaluate the degree of agreement between different methods (devices, instruments or observers) used to measure the same characteristic. Thus, the closeness between the measures of the methods must be evaluated. Different procedures for assessing agreement with continuous measurements have been proposed and these can be classified under two terms [1]: (1) unscaled summary indices based on absolute differences; and (2) scaled summary indices which translate absolute differences into more meaningful values ranging between -1 (perfectly reversed agreement) and 1 (perfect agreement), where 0 indicates no agreement.

Scaled indices have probably been the most widely used, especially the intraclass correlation coefficient [2-4] (ICC) and the concordance correlation coefficient [5] (CCC). Both ICC and CCC indices have recently been evaluated and compared in many studies [6-8], and have been shown to provide two different expressions of one common index. However, when conducting an agreement analysis it should be remembered that these scaled indices depend on the covariance between measurement devices [9], as the resulting estimates can vary depending on the possible range of values of the measurement instrument under consideration. Another consequence of this covariance dependency is that the indices might be overestimated if potential confounding variables are not taken into account [8].

Among unscaled procedures, the total deviation index (TDI) describes a boundary such that a majority percent of the differences in paired measurements are within the boundary [10,11], i.e. a probability interval. The advantage of the TDI against scaled measures such as CCC is that it does not depend on the data range and therefore it avoids the inconvenience of not taking into account potential covariates that explain between-subject variation. However it must be noted that as in the CCC case the TDI will depend on covariates explaining within-subject variation. A further advantage is that it has a straightforward interpretation since it results in the same measurement scale as that of the variable considered for agreement purposes.

Several methods for inference about the TDI estimate have been proposed. To calculate the index Lin [10] derived the cumulative probability function of the square of the paired-measures difference variable, which is assumed to follow a non-central chi-squared distribution. He argues that inference about the estimate of the resulting equation is cumbersome, and he thus derives a further approximation with more desirable properties based on the asymptotic theory of the mean squared deviation (MSD) [10]. Lin et al. [12] extended the method to deal with repeated measures. Due to the positive skewness of the resulting TDI estimates, when performing inferences the natural log transformation of the estimate is used. This approximation has been shown to conclude satisfactory agreement when mean differences between two measurement devices are small, but it can be conservative when the relative bias square value is unreasonably large and when the coverage probability is large (0.95). Choudhary and Nagaraja [13] proposed an upper bound for the estimate of Lin's resulting TDI equation for the case of no repeated measures derived from an exact test. As the exact test method needs to maximise an integrated equation with no closed form, numerical computations are required to implement it; as the authors acknowledge, these may not be readily available in practice, so they also propose a closed-form approximation.

Choudhary [14] subsequently extended the method based on the asymptotic distribution of the logarithm of Lin's TDI proposal to deal with repeated measures. He argues that this method performs well with large sample sizes and proposes a modified version for smaller sample sizes based on a bootstrap approach. Recently, Quiroz and Burdick [15] also derived a method for inference about the TDI estimate when dealing with repeated measures for the two methods that are paired over time, and fit the data using an ANOVA model. They then construct generalised confidence intervals about the TDI estimate that are based on replacing parameters involved in Lin's [10] TDI expression with generalised pivotal quantities. The generalised confidence intervals are constructed via Monte Carlo simulations and have been shown to perform well in a wide range of scenarios, including those with either small, moderate or large sample sizes. Here we propose a technical simplification for inference about the TDI estimate based on a closed approach. We first estimate the TDI by finding the appropriate probability interval of the distribution of the paired-measures difference variable. Therefore, a natural way of making inferences about this TDI estimate is to derive its tolerance interval (TI). This procedure offers a straightforward approach as the theory and methods about TI for normal populations are well established [16-18].

The article is structured as follows: in the methods section the TDI is defined and Lin's [10] first approach is described. A brief description of two current closed approaches for inference about the TDI estimate is subsequently given. Thereafter, a probability interval approach is defined to obtain an alternative expression of the TDI estimate. This approach is also used to derive estimates of the inter- and intra-method [12,19] measures of agreement when more than one reading per subject is available. Based on the probability interval approach a direct inference method about this estimate is derived via the TI. Lastly, in this section we also describe how one may utilize the TI approach to perform inference for the computation of the coverage probability, an agreement measure related to the computation of the TDI. In the results section we illustrate the methodology by using it to evaluate agreement between a manual and an automatic blood pressure device. In this example we point out the independence of the TI method from the effect of the between-subject variation, as compared with other scaled methods such as the CCC, whose covariate adjustment that explains between-subject variation modifies the resulting agreement value. We will also describe and report our simulation study procedure for evaluating the performance of the method and compare it to already established methods. A discussion and concluding remarks are given at the end of the manuscript.

## Methods

### Definition

Suppose a continuous variable is measured by two different devices *m *times each from *n *different subjects. Therefore, the data can be fit using the following mixed model [12,14]:(1)

where *y*_*ijl *_is the *l*th measurement from subject *i *by device *j*, with *i *= 1, ..., *n*, *j *= 1, 2, and *l *= 1, ..., *m*. *δ *is the vector of fixed effects parameters common to both devices and **x**_*ijl *_is the corresponding row of the design matrix for covariates, *β*_*j *_is the fixed device effect, *α*_*i *_is the individual random effect assuming that *α*_*i *_~ *N*(0, ), *γ*_*ij *_is the individual-method interaction random effect with *γ*_*ij *_~ *N*(0, ) and *e*_*ijl *_is a random error assuming that *e*_*ijl *_~ *N*(0, ) and is independent of any other component of the mixed model. If the error variability differs across devices, then *e*_*ijl *_~ *N*(0, ).

Lin [10] defined the TDI as a boundary, *κ*_*p*_, which captures a large proportion, *p*, of paired-measurement differences from two devices or observers within the boundary, i.e., the value of *κ*_*p *_that yields *P*(|*D*| <*κ*_*p*_) = *p*, where *D *is the paired-differences variate. Under the assumption of the mixed model in (1), *D *is the paired-differences variate based on any one of the replicates, *D *= (*y*_*ijl *_- *y*_*ij'l'*_), and hence *κ*_*p *_based on *D *is actually known as the total-TDI for evaluating total agreement [12]. It is shown that the distribution of *D *is then *D *~ *N*(*μ*_*D*_, *σ*_*D*_) with *μ*_*D *_= *β*_*j *_- *β*_*j' *_and , or in the case of different error variances between devices .

When more than one reading per subject given by device *j *is available, one might be interested in measuring, in addition to the total agreement, the inter- and intra-method agreement [12,19]. Intra-method indices are used to measure the agreement among the multiple readings obtained from the same device [12]. This agreement measure is useful when ones wishes to evaluate the reproducibility or repeatability of a specific device. To evaluate intra-method agreement, differences between replications from the same individual given by the *j *- *th *device are used and, therefore, under the assumption of the mixed model in (1): (*y*_*ijl *_- *y*_*ijl'*_) ~ *N*(0, ) with  = 2. Inter-method agreement is used to measure the agreement among different devices based on the average of their multiple readings [12]. If we denote , under the assumption of the mixed model in (1), the inter-method agreement can be evaluated by the following distribution: (*y*_*ij*. _- *y*_*ij'*._) ~ *N*(*μ*_*D*_, ), where *μ*_*D *_= *β*_*j *_- *β*_*j' *_and , or in the case of different error variances between devices, .

The first formal definition [10] of the TDI for the case of one single reading from each device for each subject, i.e when *m *= 1, was based on the distributional assumption of the square transformation of the paired-measurement difference variable:(2)

where *F*^-1 ^is the inverse of the cumulative probability function of |*D*|, and *χ*^2(-1)^(·) is the *p*-th percentile of a non-central chi-square distribution with 1 degree of freedom and non-centrality parameter . Even though equation (2) was defined for the case of one single reading, one can apply the mixed model (1) to accommodate replicated readings from each device for each subject and use the model parameter estimates to obtain estimates of *μ*_*D *_and  and, furthermore, compute the TDI estimate by plugging in these estimates [14]. However, and as Lin [10] argued, inference about this *κ*_*p *_estimate is cumbersome, and he therefore proposed a further approximation based on the mean squared deviation (MSD):(3)

where *ε*^2 ^= *E*(*y*_*ijl *_- *y*_*ij'l'*_)^2^, *z*_(1 + *p*)/2 _is the (1 + *p*)/2 - *th *percentile of the standard normal distribution and |·| is the absolute value.

### Current approaches for inference about the TDI estimate

There are two already existing closed procedures for inference about the TDI estimate that consider repeated measures taken by each of the two devices with multiple readings being compared. The first approach was defined by Lin et al. [12] where the authors expressed the TDI approximation based on the MSD, as in (3), which under the assumption of the mixed model in (1) the MSD becomes , and therefore , where  = (*β*_*j *_- *β*_*j'*_)^2^/2 is defined as the variance between the two devices. Furthermore, the generalized estimating equations (GEE) approach [20] is used to obtain the model parameter estimates in (1). Since this TDI estimate is positively skewed [11,12] the authors use the log transformation to form inference and the delta method is applied to obtain the variance of the resulting TDI estimate.

The second approach was defined by Choudhary [14] where the author proposes to use the maximum likelihood estimation (MLE) procedure to obtain model parameter estimates in (1) and, furthermore, compute the TDI estimate by simply plugging the MLE estimates of *μ*_*D *_and  in (2). The author argues that the distribution of this MLE estimate of the TDI approach normality more quickly on the log scale, especially when the sample size is small. Based on this assumption the delta method is used to obtain the variance of the log-transformed TDI estimate.

Both approaches for inference about the TDI estimate are based on the delta method, which means that one should first find the partial derivatives of the log transformed TDI with respect to the model parameters used to obtain the expression of the TDI and then find the inverse of the information matrix for the fitted model.

### TDI as a probability interval

Consider the TDI definition, which sets a boundary such that a majority *p *percent of the differences in paired observations are within the boundary: *P*(|*D*| <*κ*_*p*_) = *p*. This is the same as finding *κ*_*p*_, such that *P*(-*κ*_*p *_<*D *<*κ*_*p*_) = *p*. Thus, [-*κ*_*p*_; *κ*_*p*_] defines the probability interval of *D *centered at 0, regardless of the mean value of *D*. Since *D *is assumed to behave as a normal distribution with mean *μ*_*D *_and standard deviation *σ*_*D*_, one can derive *κ*_*p *_using standard methods for computing probability intervals:(4)

where , *s *= 1, 2, are the *p*_*s*_-*th *percentile of the standard normal distribution, such that  = -2*μ*_*D*_/*σ*_*D *_-  and *p*_1 _- *p*_2 _= *p*. Therefore, one can find *p*_1 _by using its link with *p*:(5)

where Φ(·) is the cumulative standard normal distribution.

However, *p*_1 _cannot be found in a closed form using equation (5), so a recursive algorithm is required. We propose to use a modified version of the binary search algorithm [21] to find *p*_1 _and, furthermore, to compute *κ*_*p *_using (4).

Typically, the binary search algorithm is used to search in an ordered array for a single element by repeatedly dividing the array in half. Here we translate the ordered array into the interval [*low*; *high*], which means that *low*(*high*) is the lowest(highest) value that *p*_1 _can take. Now, since equation (5) has a single solution for *p*_1 _in the interval [*p*; 1], one can repeatedly halve the interval in an adequate manner to find the optimum for *p*_1_. Therefore, the algorithm is implemented as follows:

1. begin with the interval [*low *= *p*; *high *= 1];

2. calculate the midpoint of the interval *mid *= (*low *+ *high*)/2;

3. if the left-hand side of equation (5) for *p*_*i *_= *low *is greater than *p *up to a tolerance bound *δ *(i.e., ), then recalculate the interval [*low *= *mid *+ *δ*; *high *= 1]; if it is lower than *p *up to a tolerance bound *δ *(i.e. ), then recalculate the interval [*low *= *p*; *high *= *mid *- *δ*];

4. repeat steps 2-3 until convergence, i.e. until the solution for *p*_1 _in (5) is *p *- *δ *< Φ(*z*_*p*1_) - Φ(-2*μ*_*D*_/*σ*_*D *_- ) <*p *+ *δ*.

The advantage of using this iterative algorithm is its speed, as it converges on the true value of *p*_1 _in a logarithmic order of growth.

This probability interval procedure ensures that the lower bound of the interval is symmetric with the upper bound about 0; therefore it is only necessary to search for one of the interval's two limits, as the other is symmetrical about zero.

We propose to use the restricted maximum likelihood estimation (REML) method [22] to obtain the model parameter estimates in (1) and furthermore compute the TDI estimate based on probability intervals by plugging in the REML estimates of *μ*_*D *_and *σ*_*D *_in (4).

We must note that this resulting estimate of the TDI yields the same estimate as that directly computed from equation (2) using the sample counterparts, however as we will illustrate in subsequent sections this binary search algorithm is necessary to compute our proposal for the upper confidence limit of TDI.

### Intra- and inter-method TDI

The TDI based on probability intervals can also be used to assess inter- and intra-method agreement measures.

To evaluate intra-method agreement, we use the difference between two replications for the *i *- *th *individual given by the *j *- *th *device and, therefore, under the assumption of the mixed model in (1): (*y*_*ijl *_- *y*_*ijl'*_) ~ *N*(0, ). Now, since the probability distribution is centered at 0, the TDI can easily be derived via a probability interval:(6)

In fact, this resulting approach corresponds to the ISO definition of the standard way of measuring the reproducibility or repeatability of a device for the specific case where the 95^*th *^percentile point of the standard normal is used for *z*_(1+*p*)/2 _[23].

If the variability differs across devices one should then obtain two different intra-method agreements as , with *j *= 1, 2.

Inter-method agreement can be evaluated by the following distribution: (*y*_*ij*· _- *y*_*ij'*_) ~ *N*(*μ*_*D*_, ), and therefore:(7)

where *p*_1 _is found by using the modified binary search algorithm detailed previously.

### A tolerance interval (TI) for inference about the TDI estimate

Our proposal for inference about the TDI estimate is based on tolerance intervals (TI), provided that we estimate the TDI by deriving the limits of a probability interval that contains a specified *p*-proportion of the resulting estimated normal distribution.

Now, since we use estimates of the normal distribution parameters of *D *instead of using true values, *κ*_*p *_is obtained by replacing *μ*_*D *_and *σ*_*D *_by their REML estimate counterparts derived from model (1) in expression (4):(8)

Therefore, a natural way of making inference about  is to compute a one-sided tolerance interval [17,18] that covers the *p*_1_-percent of the population from *D *with a stated confidence. This is analogous to computing a one-sided confidence interval for the limit that defines the one-sided probability interval which contains the *p*_1_-percent of the population of the estimated distribution of *D*, where *p*_1 _is found using the modified binary search algorithm.

Thus, let *T *be the *studentized *variable of . It is shown, then, that *T *follows a non-central Student-*t *distribution with non-centrality parameter :(9)

where *N *= 2 × *n *× *m *is the total possible paired-measurement differences between the two devices. The degrees of freedom, *ν*, are derived from the residual degrees of freedom. We have adopted here the conservative situation, as ANOVA (analysis of variance) philosophy (see for example Searle et al. [24]), where all fixed and random effects consume degrees of freedom and, therefore, *ν *= 2 × *n *× (*m *- 1). If there is no individual-device interaction, then the degrees of freedom are *ν *= 2 × *n *× *m *- (*n *+ *m *- 1). However one can also adopt a less restrictive position and consider that the random effects do not consume degrees of freedom and in that case *ν *= 2 × *n *× *m *- 2. In situations where the variability differs across devices, the error variance of the difference between paired measurements is obtained as a linear combination of the two residual variance estimates, so the degrees of freedom can be achieved more efficiently using the Satterthwaite adjustment [25].

One can therefore construct an upper bound (UB) for the TDI estimate by using the following *cf *= (1 - *α*) × 100% one-sided TI, where *α *is the type I error rate:(10)

This TI corresponds to the exact one-sided tolerance interval for at least *p*_1 _proportion of the population defined by Hahn [17] and Hahn and Meeker [18].

For computing the above TDI approach, a SAS macro and an R function are available in additional file 1. The same rationale is used to construct an upper bound for the intra- and inter-method TDI estimates derived from plugging the REML estimates from expression (1) into expressions (6) and (7), respectively. The upper bounds are constructed as in the following expressions:(11)(12)

One can also adopt the TI defined in expressions (10), (11) or (12) to perform a hypothesis test if the interest is to ensure that at least *p*-percent of the absolute differences between paired measurements are less than a predetermined constant *κ*_0_. Therefore, the null hypothesis would be defined as in Lin [10], and take the form

and *H*_0 _would be rejected with a type I error *α *if

where one should use the appropriate  and  if the hypothesis test is constructed to evaluate total-, intra- or inter-method TDI.

### Coverage probability (CP)

Another user friendly measure of agreement which is related to the computation of the TDI is the so called coverage probability (CP) [11,12]. The CP describes the proportion captured within a pre-specified boundary of the absolute paired-measurement differences from two devices, i.e., the value of *p*_*κ *_such that *P*(|*D*| <*κ*) = *p*_*κ*_. Therefore one can find *p*_*κ *_for a specified boundary *κ *using standard methods for computing probability quantities under normal assumptions [11]:(13)

and to obtain a CP estimate, *p*_*κ *_can be computed by replacing *μ*_*D *_and *σ*_*D *_by their REML estimate counterparts derived from model (1).

As with the TDI, the CP criterion can also be translated into a hypothesis test specification. In this case the interest is to ensure that a specified boundary of the absolute paired-measurement differences captures at least a predetermined proportion, *p*_0_:

The proposed TI method for inference about the TDI can be utilized to perform inferences about the CP estimates. From the TI in (10) it follows that(14)

Now *κ *is a fixed known boundary, and our interest lies in finding a lower confidence bound for the CP estimate. Thus, one can find a lower confidence bound for a non-central Student-*t *proportion with confidence level 1 - *α *by searching the non-centrality parameter, that depends on  and hence on *p*_*κ*_, that satisfies(15)

and once the non-centrality parameter  is achieved, a lower bound about the proportion *p*_*κ *_is found using equation (5), *p*_*κ *_= Φ() - Φ(-2*μ*_*D*_/*σ*_*D *_- ).

However, the non-centrality parameter cannot be found in a closed form, so one may use again a modified version of the binary search algorithm as follows:

1. begin with the interval [*low *= 0; *high *= 1], as *p*_*κ *_is bounded by the interval (0,1);

2. calculate the midpoint of the interval *mid *= (*low *+ *high*)/2 and compute the difference ;

3. if *d *is greater than 0 up to a tolerance bound *δ *(i.e., ), then recalculate the interval [*low *= *mid *+ *δ*; *high *= 1]; if it is lower than 0 up to a tolerance bound *δ *(i.e. ), then recalculate the interval [*low *= 0; *high *= *mid *- *δ*];

4. repeat steps 2-3 until convergence, i.e. until *d *satisfies .

## Results

### Case-example: blood pressure device data

The method proposed here to assess agreement using the TDI measure will now be illustrated in a real case example. We will also show that the independence of the method from the effect of the covariance between devices (between-subject variability) constitutes an advantage of unscaled over scaled indices such as the CCC.

A sample of 384 subjects was collected and measures of systolic blood pressure were taken via two instruments: a handle mercury sphygmomanometer device and an OMRON 711 automatic device. The systolic blood pressure was measured twice by each instrument. Gender, age and heart rate were also recorded as covariates.

**Figure 1:**
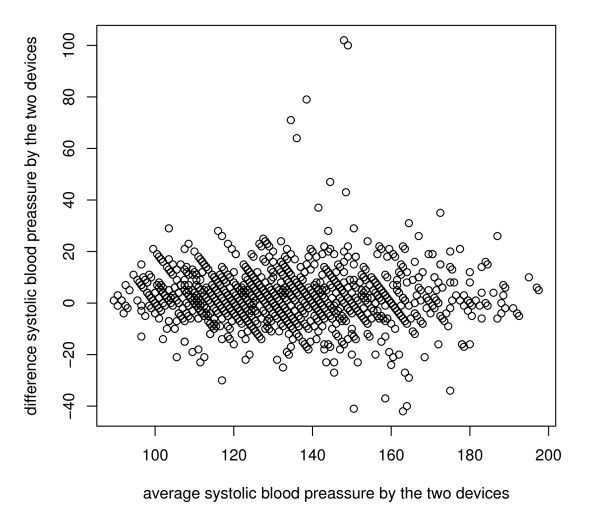
**Blood pressure device data**. Bland and Altman plot of systolic blood pressure measured using automatic device (OMRON 711) and handle device (mercury sphygmomanometer). The total possible paired-measurements are represented.

A Bland-Altman plot is shown in Figure 1. It can be seen that measurements taken from the handle sphygmomanometer tend to be discretized in round values of 10 units each, while measurements from the automatic instrument are dispersed around the range of values of the systolic blood pressure. As a result of discussions with clinical practitioners, one can assume that the manual instrument can be replaced by the automatic device if a large proportion of paired-measurement differences are within a boundary of 10 mmHg. Under this hypothesis the TDI measure is appropriate for making such a decision.

We first fit the mixed model with 'measurement device' as the fixed effect and 'individual' and 'individual-device interaction' as random effects (Model 1, Table 1) and thereafter we excluded the 'individual-device interaction' in a second model which produced a similar fit (Akaike Information Criterions (AIC): *AIC*_*Model*1 _= 11764.83 vs. *AIC*_*Model*2 _= 11760.83). The resulting estimates of  = 10.283 and  = 2.174 are used to obtain the TDI estimate. Assuming that the TDI should contain at least a proportion of 0.90 of the paired measurements between devices, the TDI estimate was 17.29 and its corresponding one-sided 95% TI was 17.93 (TDI estimates for proportions of 0.80, 0.85 and 0.95 are shown in Table 2). The TI's were calculated using a tolerance bound of 1e-10 and the computed values of *p*_1 _for proportions of 0.80, 0.85, 0.90 and 0.95 were 0.864, 0.896, 0.929 and 0.963 respectively.

**Table 1:** Blood pressure device data: model parameter estimates

	Effects	Model1	Model2	Model3	Model4
**Random**	**individual**	380.187	380.187	221.396	221.391
	individual*device	1.56e-06	-	3.00e-4	-
	**error**	52.867	52.867	52.867	52.867

**Fixed**	**intercept**	133.369	133.369	84.864	84.864
		(1.029)	(1.029)	(5.061)	(5.061)
	**device**	2.174	2.174	2.174	2.174
		(0.371)	(0.371)	(0.371)	(0.371)
	**gender**	-	-	-9.496	-9.496
		-	-	(1.585)	(1.585)
	**age**	-	-	0.817	0.817
		-	-	(0.057)	(0.057)
	**heart rate**	-		0.194	0.194
		-	-	(0.069)	(0.069)

**AIC**		11764.83	11760.83	11574.54	11570.54

Restricted maximum likelihood estimates of the mixed effects models. The values of random effects are variances, whereas point estimates and standard errors (between brackets) are shown for fixed effects.

**Table 2:** Blood pressure device data: concordance measures

		Percentile	Lin		Choudhary		TI	
*p*				*UB*_95%_()		*UB*_95%_()		*UB*_95%_()
**0.80**	**Total TDI**	10	14.3	16.0	13.5	13.9	13.5	14.0
	**Intra-method TDI**	10.5	14.1	15.7	13.2	13.6	13.2	13.8
	**Inter-method TDI**	7.4	10.3	11.6	-	-	10.2	10.6

**0.85**	**Total TDI**	12	16.1	17.9	15.1	15.7	15.1	15.7
	**Intra-method TDI**	14	15.8	17.7	14.8	15.3	14.8	15.5
	**Inter-method TDI**	9	11.6	13.0	-	-	11.3	11.8
**0.90**	**Total TDI**	15	18.4	20.5	17.3	17.9	17.3	17.9
	**Intra-method TDI**	16	18.1	20.2	16.9	17.5	16.9	17.7
	**Inter-method TDI**	11	13.3	14.9	-	-	12.9	13.3

**0.95**	**Total TDI**	19	21.9	24.4	20.6	21.3	20.6	21.3
	**Intra-method TDI**	20.5	21.5	24.1	20.1	20.8	20.2	21.0
	**Inter-method TDI**	15	15.8	17.7	-	-	15.2	15.7

Percentile of the absolute difference, total, inter- and intra-method Total Deviation Indices for four different proportion sets. Results represent the TDI estimates, , based on Lin, Choudhary and our Probability Interval approaches respectively and the resulting 95% upper bounds, *UB*(), based on Lin Choudhary and our Tolerance Interval (TI) approaches.

We also applied Lin's and Choudhary's procedures described in the methods section; the second produced the same results as our TI proposal and Lin's approach resulted in more conservative estimates compared to the respective percentiles calculated from the absolute difference. Though these percentiles are naive estimates of the TDI's, they can serve as the reality check for comparing across the three methods, since we do not know the theoretical values. Based on the three methods applied, under the hypothesis of disagreement between devices, if a large proportion of absolute paired-measurement differences are above a boundary of 10 we would not reject disagreement, thus the two devices are not interchangeable.

We then entered gender, age and heart rate as covariates into the model. The inclusion of covariates in the model did not modify the parameter estimates used to calculate the TDI, i.e. the device fixed-effect and the error variance estimates, and therefore the TDI estimates as well as their 95% one-sided TI remain the same.

Finally, we also calculated the intra- and inter-method TDI containing 80%, 85%, 90% and 95% proportions, as shown in Table 2. The intra-method TDI is interpreted as the boundary at which the specified proportion of the replicated measurements are furthest from themselves. The inter-method TDI is interpreted as the boundary at which the specified proportion of the average of the replicated measurements from one device are furthest from the average of the replicated measurements of the other device. In the case example, for all four proportions specified, the intra-method TDIs are larger than the pre-specified boundary of a difference of at least 10 to ensure agreement (the difference observed between Lin's and our TI proposal is due to the estimation method of the variance components), which means that the principal problem with the total-TDI is due to the fact that the intra-individual variability is too large rather than the systematic bias. In other words, if one calibrates both devices, i.e., in the absence of bias, the devices would still not be interchangeable. Therefore a specific device for measuring the systolic blood pressure is not interchangeable with itself and it is somewhat pointless to assess agreement between these two devices since they are not repeatable within themselves.

### Simulation study

The performance of the method to evaluate agreement via the TDI estimate using probability intervals, as well as inference via the TI approach, will be assessed and compared to the two already established methods by means of a simulation study.

The scenario is based on the real case example of blood pressure device data where two measures for each of the two devices are simulated. For the sake of simplicity we assumed, as in the case-example, no interaction effect between individuals and devices. We therefore held fixed the intercept and the variance component of the individual random effect equal to the mixed-model point estimates in the original data (Table 1, Model 2), while we simulated different combinations of fixed device effects and random error variance. We first considered a device effect equal to the point estimate obtained from the original data (2.174), and then simulated two other more extreme values: a mean difference of 0 and a larger mean difference of 5. Likewise, we simulated a random error variance equal to the case-example point estimate (52.867), which gives a standard deviation of the difference between devices of 10.283, and then simulated a smaller random error variance of 16, which gives a standard deviation for the paired differences around half the value obtained in the original case-example data (5.65). Sample sizes of 20 and 100 individuals were considered. For each scenario considered, the simulation study involved generating 1000 samples of the measurements vector with the particular structure. The algorithm used to generate the *s *- *th *(*s *= 1, ..., 1000) sample can be summarised in the following steps:

1. set *δ *and  and set values for *β *and ;

2. generate each measurement data vector **y**^*s *^from the multivariate normal distribution *MV N*(**X**(*δ*, *β*)^*t*^, **V**(, ));

3. fit the mixed model for each data set using GEE when Lin's approach is applied, MLE for Choudhary's approach and REML for our proposal.

Note that the parameters of the multivariate normal distribution in step 2 come from the matrix notation of the mixed model described above, where **X **is the design matrix of the fixed effects and **V **is the block-diagonal total variance-covariance matrix with diagonal elements equal to  and off-diagonal elements equal to .

The TDI point estimate via probability intervals and their corresponding TI were computed for each case, with a tolerance equal to 1.0e-4, as well as Lin's and Choudhary's proposals.

The accuracy of the TDI estimate was calculated in order to determine whether the TI was reliable. Thus,

we calculated the mean of the TDI estimates and the mean square error, *MSE *= *E*( - *κ*_*p*_)^2^, where the *actual κ*_*p *_is calculated using Lin's [10] definition, as in equation 2.

To evaluate the performance of the TI approach for inference about the TDI estimate we analyzed the empirical confidence (EC) of the TI as , where *I*^*s *^= 1 if *κ*_*p *_is within the TI. The same rationale was applied for the two other established methods.

Since the distribution property of a TDI estimate has been shown to be log-normal [11,12], the mean and MSE are computed based on the log transformation of the TDI estimates, and the EC are directly computed from the upper limits of the log transformed TDI estimates.

Table 3 shows that good point estimates of the TDI are obtained in all of the simulated combinations; however, the small MSEs found increase in line with the difference between devices, the standard deviation of the differences, and the proportion of the population that should be contained within the TDI boundary increase. The fact that the MSE is lower for larger sample sizes indicates the consistent asymptotic properties of the probability interval estimation approach. It is also shown that in simulations based on no difference between devices, a systematic, slight overestimation is found in TDI point estimates.

**Table 3:** TDI simulation results

				Mean			MSE × 1000			EC		
	*p*	n	log(*κ*_*p*_)	Lin	**Ch**.	PI	Lin	**Ch**.	PI	Lin	**Ch**.	TI
*μ*_*D *_= 0	0.80	20	1.981	2.014	1.977	1.985	10.409	8.492	8.496	95.7	94.7	98.5
*σ*_*D *_= 5.65		100		1.997	1.980	1.981	2.040	1.617	1.615	97.4	94.8	98.5
	0.85	20	2.097	2.131	2.093	2.102	10.425	8.491	8.498	95.7	94.7	98.3
		100		2.133	2.096	2.098	2.048	1.616	1.615	97.4	94.8	98.3
	0.90	20	2.231	2.264	2.227	2.235	10.381	8.496	8.491	95.7	94.7	97.9
		100		2.246	2.229	2.231	2.027	1.618	1.615	97.3	94.4	97.9
	0.95	20	2.406	2.439	2.402	2.410	10.399	8.495	8.491	95.7	94.7	97.6
		100		2.421	2.405	2.406	2.036	1.617	1.615	97.3	94.7	97.6

*μ*_*D *_= 2.174	0.80	20	2.052	2.076	2.047	2.054	10.286	9.216	9.135	95.2	94.9	97.4
*σ*_*D *_= 5.65		100		2.061	2.048	2.050	2.167	1.983	1.971	95.5	93.4	95.4
	0.85	20	2.167	2.192	2.161	2.169	10.347	9.109	9.030	95.2	94.9	96.9
		100		2.178	2.163	2.165	2.192	1.961	1.951	95.7	93.3	95.0
	0.90	20	2.300	2.326	2.293	2.300	10.364	8.998	8.902	95.3	94.8	96.6
		100		2.311	2.296	2.297	2.199	1.943	1.930	95.8	93.1	94.2
	0.95	20	2.473	2.501	2.465	2.472	10.487	8.834	8.735	95.6	94.9	96.2
		100		2.486	2.469	2.470	2.253	1.904	1.892	96.1	93.3	93.8

*μ*_*D *_= 5	0.80	20	2.287	2.281	2.276	2.281	8.736	9.211	9.061	91.9	94.6	91.0
*σ*_*D *_= 5.65		100		2.278	2.286	2.287	1.954	1.890	1.886	91.9	94.7	92.6
	0.85	20	2.391	2.398	2.378	2.383	8.748	8.658	8.505	93.6	95.0	91.4
		100		2.395	2.390	2.390	1.890	1.751	1.747	95.3	94.5	92.8
	0.90	20	2.508	2.531	2.495	2.500	9.231	8.114	7.965	94.9	94.6	91.9
		100		2.528	2.507	2.508	2.272	1.616	1.613	97.7	94.5	92.8
	0.95	20	2.662	2.706	2.647	2.652	10.660	7.624	7.463	97.2	94.1	92.0
		100		2.703	2.660	2.661	3.569	1.485	1.481	99.1	94.4	93.2

*μ*_*D *_= 0	0.80	20	2.579	2.618	2.581	2.589	10.282	7.911	8.011	97.1	96.7	99.1
*σ*_*D *_= 10.28		100		2.595	2.577	2.579	2.121	1.647	1.639	97.2	94.3	98.5
	0.85	20	2.695	2.734	2.697	2.705	10.301	7.911	8.014	97.1	96.7	98.7
		100		2.711	2.693	2.695	2.128	1.642	1.639	97.2	94.4	98.2
	0.90	20	2.828	2.868	2.830	2.839	10.327	7.912	8.018	97.1	96.7	98.3
		100		2.844	2.827	2.828	2.139	1.641	1.640	97.2	94.4	97.8
	0.95	20	3.003	3.043	3.006	3.014	10.349	7.911	8.020	97.1	96.7	98.0
		100		3.020	3.002	3.004	2.148	1.640	1.640	97.2	94.5	97.5

*μ*_*D *_= 2.174	0.80	20	2.601	2.627	2.593	2.601	9.833	8.595	8.502	95.3	95.2	97.9
*σ*_*D *_= 10.28		100		2.613	2.597	2.599	2.171	1.819	1.809	96.3	93.5	96.9
	0.85	20	2.717	2.743	2.709	2.717	9.846	8.567	8.473	95.3	95.3	97.8
		100		2.729	2.713	2.715	2.177	1.816	1.806	96.3	93.6	96.9
	0.90	20	2.850	2.877	2.842	2.849	9.864	8.529	8.435	95.4	95.2	97.3
		100		2.862	2.847	2.848	2.185	1.812	1.802	96.3	93.6	96.4
	0.95	20	3.025	3.052	3.016	3.024	9.879	8.848	8.379	95.5	95.3	97.1
		100		3.038	3.022	3.023	2.192	1.807	1.797	96.4	93.7	95.8

*μ*_*D *_= 5	0.80	20	2.689	2.706	2.682	2.689	9.727	9.684	9.550	93.6	94.1	95.8
*σ*_*D *_= 10.28		100		2.698	2.688	2.689	2.103	1.994	1.988	95.8	93.8	95.1
	0.85	20	2.803	2.823	2.795	2.802	9.810	9.444	9.313	94.0	94.2	95.7
		100		2.814	2.802	2.803	2.147	1.947	1.942	95.9	94.0	94.5
	0.90	20	2.934	2.956	2.925	2.932	9.907	9.175	9.037	94.0	94.4	95.2
		100		2.947	2.933	2.934	2.204	1.890	1.886	96.0	93.8	94.0
	0.95	20	3.105	3.131	3.095	3.102	10.112	8.826	8.684	94.1	94.1	94.7
		100		3.123	3.104	3.105	2.335	1.812	1.808	96.5	94.1	93.6

Simulation results. log(*κ*_*p*_) refers to the actual log-transformed Total Deviation Index (TDI) simulated, whereas Mean and MSE correspond to the mean value and mean squared error of the log-transformed TDI estimates from each of the scenarios considered, based on Lin, Choudhary and our Probability Interval (PI) approaches respectively. EC refers to the empirical confidence based on the 95% confidence level based on Lin, Choudhary (Ch.) and our Tolerance Interval (TI) upper bounds of the log-transformed TDI estimates respectively.

ECs, for each scenario combination, evaluating the 95% nominal coverage of the TI approach, are shown in Table 3. The results show that the TI approximation produces accurate coverage rates which are reasonably close to the nominal coverage. It should be highlighted that combinations with higher EC are those based on a mean difference between devices of 0. This is a result of the systematic overestimation of the TDI. At the other extreme, simulations based on mean differences of 5 and small standard deviation for paired differences show a EC lower than the desired 95% nominal coverage, although this is only observed in cases with small sample sizes. However, the coverage rates do increase towards the nominal coverage with larger sample sizes.

**Figure 2:**
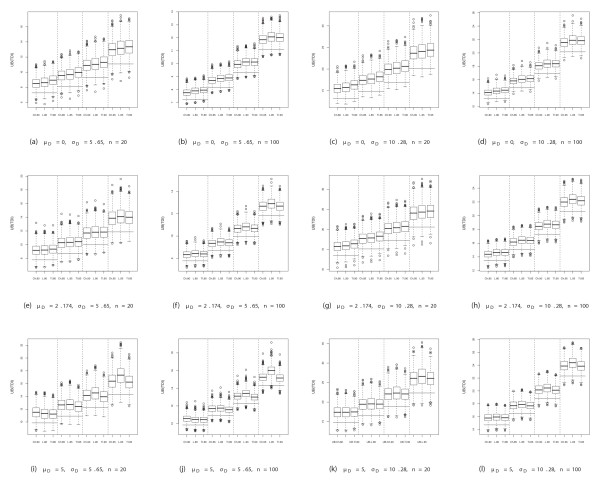
**Simulation results: upper bounds of the TDI estimates**. Boxplots of the upper bounds of the TDI estimates (UB(TDI)) based on Choudhary (Ch), Lin (L) and our Tolerance Interval (TI) approaches for each of the scenarios considered. Horizontal lines refer to the actual TDI simulated based on the four different proportion sets (80%, 85%, 90% and 95%).

Simulation results of the current approaches are also shown in Table 3. Choudhary's proposal seems to produce the most stable results in terms of EC in all the scenarios simulated, however the range of the estimated TDI upper bounds are very similar to our TI approach (Figure 2), the difference is only seen in scenarios simulated with a mean difference of 0, even though the boxplots in these situations are shifted up in the worst case by no more than 0.5 units. Lin's approach results in intermediate values between Choudhary's results and our TI proposal results for these specific scenarios. However Lin's upper bounds of the TDI estimates seem to increase in line with larger mean difference, stated proportions and sample size (Figure 2). This issue is in accordance with the results found in [12] where the authors also recognized that their approximate TDI can be conservative when the relative bias square value is unreasonably large and when the coverage probability is large (>0.9).

## Discussion

With the aim of assessing agreement between two devices of continuous measurements via the total deviation index (TDI), the present study evaluated the performance of a simplified technical approximation of the TDI based on probability intervals and a tolerance interval (TI) approach for inference about the resulting TDI estimate.

The parameters involved in the TDI are obtained from a linear mixed effects model estimated via REML. The linear mixed model has the advantage of its flexibility that allows adapting the model to the data features as replicates.

Several methods have been implemented for making inferences about the TDI estimate [10,12-15,26]. However, since all these methods are based on the square transformation of the paired-measures difference variable, which makes exact inference about the resulting estimate difficult, inference is carried out using analytical approaches or methods based on Monte Carlo simulations.

Bland and Altman [27,28] also defined an unscaled agreement index known as limits of agreement, which is similar to the TDI. The authors derive the limits as boundaries, such that a majority percent of the paired-measurement differences fall within the boundaries using a probability interval, and thereafter they derive a TI for inference about the limits. However, since the intervals are constructed to be symmetrical about the mean difference, and not symmetrical about 0, the TDI could be constructed by taking the maximum of the absolute value among lower and upper limits: *max*(|*L*_*low*_|, |*L*_*upp*_|). Conversely, the initial percent that is assumed to fall within the boundaries would result in a larger proportion thereafter. The proposal introduced here corrects this fact and, as a result, the "effective length" of the interval is shortened.

We have also shown in the present study how the proposed method can be used to compute bounds for the coverage probability (CP). As the computation of CP is related to the computation of the TDI, the performance of the CP bound behaves very similarly to the TDI bound (results from a simulation analysis are shown in additional file 2).

Although our proposal has been shown to provide accurate empirical confidences it does tend to overestimate the nominal confidence level slightly, especially for small differences between devices. In terms of hypothesis testing this means that the type I error will be smaller than the desired nominal rate in this particular scenario. In agreement assays were the aim lies in evaluating if one currently used device can be replaced by another one, as in our case example, this might be a benefit since it means that replacing a good device by a bad device is very unlikely. This issue was already detected by Westlake [29], who proposed a modification of the conventional confidence interval method to obtain symmetrical confidence intervals around 0 for bioequivalence trials. The limits of the confidence intervals were constructed in the same manner as proposed here to obtain the probability intervals to estimate the TDI. Westlake demonstrated that the confidence level constructed in this way is 100% for a mean difference of 0 and larger sample size, decreasing monotonically to the desired nominal confidence as the difference tends to infinity. A limitation of our proposal is seen when the mean difference between devices is large compared to the standard deviation and the sample size is small, in these situations the type I error will be slightly larger than the desired nominal rate.

## Conclusions

Finally, we would like to highlight that the method proposed here is straightforward since the TDI estimate is derived directly from a probability interval of a normally-distributed variable in its original scale, without further transformations. Thereafter, a natural way of making inferences about this estimate is to derive the appropriate TI. The expression of our TI proposal corresponds to the exact one-sided TI defined by Hahn in 1970 [17] for at least a pre-specified proportion of a normally distributed population, with the particularity that the specified proportion is found using a search algorithm to ensure the confidence bounds be symmetrical about 0. This procedure has been shown to provide accurate coverage rates, even though it is slightly more conservative than Lin's and Choudhary's approaches in the case of no systematic bias, which both show results closer to the nominal confidence level. However the TI results in these situations are reasonably close to those given by these other established methods. At the other extreme, when there is a large bias compared to the standard deviation and the sample size is small, the empirical confidence is slightly smaller than the stated nominal confidence, but again the TI results are very close to those given by Choudhary's proposal which appears to be the most stable approach in terms of empirical coverage in this situation. The advantage of our proposal is that constructions of TI are implemented in most standard statistical packages, thus making it simpler for any practitioner to implement it to assess agreement.

## Competing interests

The authors declare that they have no competing interests

## Authors' contributions

GE and JLC conceived, designed and performed the analysis. GE, JLC and CA were responsible of the interpretation of the results and drafting the paper. All authors read and approved the final manuscript.

## Pre-publication history

The pre-publication history for this paper can be accessed here:

http://www.biomedcentral.com/1471-2288/10/31/prepub

## Supplementary Material

Additional file 1 **Software codes**. Description of a SAS macro and an R function developed to compute the TDI estimate and upper confidence bound. Click here for file

Additional file 2 **2CP simulation results**. Simulation results about the performance of the coverage probability (CP) index. Click here for file
