# Supplementary material for: The Total Deviation Index estimated by Tolerance Intervals to evaluate the concordance of measurement devices
Source: BMC Med Res Methodol. 2010 Apr 8;10:31. doi: 10.1186/1471-2288-10-31 (PMC2859350; doi:10.1186/1471-2288-10-31)
Supplement: Additional file 1 — Software codes. Description of a SAS macro and an R function developed to compute the TDI estimate and upper confidence bound. [file 1471-2288-10-31-S1.PDF]

SAS macro that computes the TDI estimate and it's upper confidence bound

```
%macro tdi_ti(dataset,m,p,alpha,tol,int=0);

* Measurement variate must be named "y";
* Subject effect must be named as "id";
* Device effect must be named as "met";
* m is the number of measures by subject and device;

%if (&int ne 0) and (&int ne 1) %then %do;
       %put 'int must be 0 (Model with no interaction) or 1 (Model with interaction)';
       %goto exit;
%end;


* Model with no interaccion;
%if (&int=0) %then %do;
       proc mixed data=&dataset;
       class id met;
       model y=met /solution;
       random int /subject=id;
       lsmeans met /pdiff;
       ods output covparms=table1;
       ods output diffs=table2;
       ods output classlevels=table3;
       run;

data temp;
set table1 (where=(CovParm='Residual') drop=subject rename=(estimate=se));
set table2  (keep=estimate rename=(estimate=md));
set table3 (drop=values where=(class='id') rename=(levels=ns));
drop covparm class;
sd=sqrt(2*se);    * Standard deviation of the difference;
df=2*ns*&m-(ns+&m-1);
%end;

* Model with interaccion;
%if (&int=1) %then %do;
       proc mixed data=&dataset;
       class id met;
       model y=met /solution;
       random int met /subject=id;
       lsmeans met /pdiff;
       ods output covparms=table1;
       ods output diffs=table2;
       ods output classlevels=table3;
       run;

data temp;
set table1 (where=(CovParm='Residual') drop=subject rename=(estimate=se));
set table1 (where=(CovParm='met') drop=subject rename=(estimate=sg));
set table2  (keep=estimate rename=(estimate=md));
set table3 (drop=values where=(class='id') rename=(levels=ns));
drop covparm class;
sd=sqrt(2*(sg+se));    * Standard deviation of the difference;
df=2*ns*(&m-1);
%end;


data temp; set temp;

* Computing TDI;
nc=md/sd;
tdi=sd*sqrt(cinv(&p,1,(nc**2)));

* Computing p1 using binary search algorithm;
low = &p; high = 1;
do while ((high-low) >&tol);
       mid=(low+high)/2;
       zp=probit(mid);
```

```
       pest=CDF('NORMAL',zp)-CDF('NORMAL',-zp-2*nc)-&p;
       if((pest<&tol) and (pest>-&tol)) then leave;
       else if pest > &tol then high=mid-&tol;
       else if pest < (-1)*&tol then low=mid+&tol;
end;
* Computing upper bound of TDI;
n=2*&m*ns;
k=1/sqrt(n)*tinv(1-&alpha,df,probit(mid)*sqrt(n));
ub=md+k*sd;
run;

proc report data=temp headline headskip split='|' nowindows;
  title "TDI and Upper Bound estimate for p=&p and alpha=&alpha";
  column md sd tdi ub;
  define md /'Mean of Dif.' format=6.2 center;
  define sd /'Std.Dev. of Dif.' format=6.2 center;
  define tdi /'TDI' format=6.2 center;
  define ub /'Upper Bound' format=6.2 center;
 run;
%exit:
%mend;
```

R function that computes the TDI estimate and it's upper confidence bound

```
# libraries needed
library(nlme)

# Measurement variate must be named "y";
# Subject effect must be named as "id";
# Device effect must be named as "met";
# m is the number of measures by subject and device;

tdi.ti<-function(dataset,m,p,alpha,tol,int=0){

       if ((int != 0) & (int != 1)){
              print("int must be 0 (Model with no interaction) or 1 (Model with
interaction)")
              break
       }

       ns <- dim(table(dades$id))    #Number of subjects

       # Model with no interaccion;
       if (int==0){
              model  <- lme(y~met,data=dades,random=~1|id,method="REML")
              md <- abs(summary(model)$coefficients$fixed[2])
              sd <- sqrt(2*(model$sigma^2))        #Standard deviation of the difference
              df <- 2*ns*m-(ns+m-1)                #Degrees of freedom
       }

       # Model with no interaccion;
       if (int==1){
              model <- lme(y~met,data=dades,random=~met|id,method="REML")
              md <- abs(summary(model)$coefficients$fixed[2])
              var.int <- getVarCov(model)[2,2]
              sd <- sqrt(2*(var.int+model$sigma^2)) #Standard deviation of the difference
              df <- 2*ns*(m-1)                      #Degrees of freedom
       }

       # Computing TDI;
       nc <- md/sd;
       tdi <- round(sd*sqrt(qchisq(p,1,ncp=(nc^2))),digits=4)

       # Computing p1 using binary search algorithm;
       low<-p
       high<-1
       while(low<=high){
              mid<-(high+low)/2
              p.est<-pnorm(qnorm(mid))-pnorm(-qnorm(mid)-2*md/sd)-p

              if((p.est<=tol)&(p.est>=-tol)){break}

              if(p.est>tol){
                     high<-mid-tol
              }
              if(p.est<(-tol)){
                     low<-mid+tol
              }
       }

       # Computing upper bound of TDI;
       n  <- 2*m*ns;
       k  <- 1/sqrt(n)*qt(1-alpha,df,ncp=(qnorm(mid)*sqrt(n)))
       ub <- md+k*sd;

       result<-list(tdi , ub)
       names(result)<-c("TDI est","TDI bound")
       return( result )
}
```
