# Supplementary material for: The Total Deviation Index estimated by Tolerance Intervals to evaluate the concordance of measurement devices
Source: BMC Med Res Methodol. 2010 Apr 8;10:31. doi: 10.1186/1471-2288-10-31 (PMC2859350; doi:10.1186/1471-2288-10-31)
Supplement: Additional file 2 — 2CP simulation results. Simulation results about the performance of the coverage probability (CP) index. [file 1471-2288-10-31-S2.PDF]

Supplementary Table - CP simulation results

Simulation results. p_κ refers to the actual Coverage Probability (CP) simulated from each of the scenarios considered. Summaries are shown for the CP point estimates, $\hat{p}_\kappa$, and CP lower bounds of the resulting estimates based on the 95% confidence level, $LB_{95\%}(\hat{p}_\kappa)$. The summaries involved are: Mean, MSE, SD and EC that correspond to the mean value of both the CP estimates and the corresponding lower bounds, the mean squared error of the CP estimates, the standard deviation of the CP lower bounds and the empirical confidence of the CP lower bounds respectively.

Table 1: CP simulation results

	κ	**n**	p_κ	$\hat{p}_\kappa$ **Mean**	$\hat{p}_\kappa$ **MSE**	$LB_{95\%}(\hat{p}_\kappa)$ **Mean**	$LB_{95\%}(\hat{p}_\kappa)$ **SD**	$LB_{95\%}(\hat{p}_\kappa)$ **EC**
$\mu_D = 0$	7.250	20	0.80	0.797	16.8e-4	0.692	0.049	98.5
$\sigma_D = 5.65$		100		0.802	16.8e-4	0.695	0.050	98.3
	8.143	20	0.85	0.848	13.8e-4	0.753	0.047	98.3
		100		0.850	2.68e-4	0.811	0.018	98.3
	9.305	20	0.90	0.896	9.73e-4	0.818	0.042	97.9
		100		0.899	1.86e-4	0.868	0.016	97.9
	11.087	20	0.95	0.946	4.67e-4	0.891	0.033	97.6
		100		0.949	0.86e-4	0.929	0.012	97.6
$\mu_D = 2.174$	7.782	20	0.80	0.798	18.5e-4	0.707	0.048	97.5
$\sigma_D = 5.65$		100		0.801	4.00e-4	0.764	0.021	95.4
	8.734	20	0.85	0.847	15.1e-4	0.767	0.046	96.9
		100		0.850	3.20e-4	0.818	0.019	95.0
	9.970	20	0.90	0.897	10.5e-4	0.830	0.041	96.6
		100		0.900	2.22e-4	0.874	0.017	94.3
	11.859	20	0.95	0.941	6.15e-4	0.891	0.033	97.7
		100		0.945	1.43e-4	0.926	0.013	98.0
$\mu_D = 5$	9.849	20	0.80	0.800	22.6e-4	0.728	0.051	91.0
$\sigma_D = 5.65$		100		0.799	4.80e-4	0.769	0.023	92.5
	10.922	20	0.85	0.850	17.5e-4	0.784	0.047	91.5
		100		0.849	3.71e-4	0.822	0.020	92.8
	12.286	20	0.90	0.900	11.4e-4	0.843	0.041	91.9
		100		0.899	2.42e-4	0.876	0.017	92.7
	14.322	20	0.95	0.949	4.87e-4	0.908	0.031	92.1
		100		0.949	1.04e-4	0.933	0.012	93.2
$\mu_D = 0$	13.178	20	0.80	0.794	16.3e-4	0.689	0.048	99.1
$\sigma_D = 10.28$		100		0.799	16.0e-4	0.690	0.049	98.8
	14.802	20	0.85	0.844	13.6e-4	0.750	0.046	98.7
		100		0.846	2.71e-4	0.812	0.019	98.2
	16.914	20	0.90	0.894	9.68e-4	0.815	0.042	98.3
		100		0.899	1.89e-4	0.868	0.016	97.8
	20.154	20	0.95	0.945	4.76e-4	0.889	0.033	98.0
		100		0.949	0.87e-4	0.930	0.012	97.4
$\mu_D = 2.174$	13.472	20	0.80	0.799	16.8e-4	0.700	0.048	97.9
$\sigma_D = 10.28$		100		0.801	3.62e-4	0.760	0.021	96.9
	15.132	20	0.85	0.848	13.7e-4	0.760	0.045	97.8
		100		0.850	2.96e-4	0.815	0.019	96.9
	17.288	20	0.90	0.898	9.54e-4	0.824	0.041	97.3
		100		0.900	2.04e-4	0.871	0.016	96.4
	20.597	20	0.95	0.948	4.48e-4	0.896	0.032	97.1
		100		0.950	0.93e-4	0.931	0.012	95.7
$\mu_D = 5$	14.714	20	0.80	0.798	19.7e-4	0.713	0.048	95.8
$\sigma_D = 10.28$		100		0.800	4.14e-4	0.764	0.021	95.1
	16.499	20	0.85	0.848	15.78e-4	0.772	0.046	95.7
		100		0.850	3.34e-4	0.818	0.020	94.5
	18.806	20	0.90	0.898	10.7e-4	0.834	0.040	95.2
		100		0.899	2.26e-4	0.874	0.017	94.0
	22.320	20	0.95	0.948	4.80e-4	0.902	0.031	94.7
		100		0.949	1.00e-4	0.932	0.012	93.4
